# Identification of distinct immune activation profiles in adult humans

**DOI:** 10.1038/s41598-020-77707-6

**Published:** 2020-11-30

**Authors:** Renaud Cezar, Audrey Winter, Delphine Desigaud, Manuela Pastore, Lucy Kundura, Anne-Marie Dupuy, Chantal Cognot, Thierry Vincent, Christelle Reynes, Catherine Dunyach-Remy, Jean-Philippe Lavigne, Robert Sabatier, Patricia Le Merre, Elisabeth Maggia, Pierre Corbeau

**Affiliations:** 1 Immunology Department, Nîmes University Hospital, Place du Pr Debré, 30029 Nîmes, France; 2 Institute of Human Genetics, CNRS-Montpellier University UMR9002, 141 rue de la Cardonille, 34396 Montpellier Cedex 5, France; 3 Institute of Functional Genomics UMR5203 and BCM, CNRS-INSERM-Montpellier University, 141 rue de la Cardonille, 34396 Montpellier, France; 4 Biochemestry Department, Montpellier University Hospital, 371 Avenue du Doyen Gaston Giraud, 34295 Montpellier, France; 5 Immunology Department, Montpellier University Hospital, 80 Avenue Auguste Fliche, 34295 Montpellier, France; 6 U1047, INSERM, Microbiology, Nîmes University Hospital, Montpellier University, Place du Pr Debré, 30029 Nîmes, France; 7 Caisse Primaire d’Assurance Maladie, 14 rue du Cirque Romain, Nîmes, France; 8 Montpellier University, 5 Boulevard Henri IV, 34967 Montpellier, France; 9 Fédération Hospitalo-Universitaire Infections Chroniques, Montpellier-Nîmes, France

**Keywords:** Immunology, Biomarkers, Medical research

## Abstract

Latent infectious agents, microbial translocation, some metabolites and immune cell subpopulations, as well as senescence modulate the level and quality of activation of our immune system. Here, we tested whether various in vivo immune activation profiles may be distinguished in a general population. We measured 43 markers of immune activation by 8-color flow cytometry and ELISA in 150 adults, and performed a double hierarchical clustering of biomarkers and volunteers. We identified five different immune activation profiles. Profile 1 had a high proportion of naïve T cells. By contrast, Profiles 2 and 3 had an elevated percentage of terminally differentiated and of senescent CD4+ T cells and CD8+ T cells, respectively. The fourth profile was characterized by NK cell activation, and the last profile, Profile 5, by a high proportion of monocytes. In search for etiologic factors that could determine these profiles, we observed a high frequency of naïve Treg cells in Profile 1, contrasting with a tendency to a low percentage of Treg cells in Profiles 2 and 3. Moreover, Profile 5 tended to have a high level of 16s ribosomal DNA, a direct marker of microbial translocation. These data are compatible with a model in which specific causes, as the frequency of Treg or the level of microbial translocation, shape specific profiles of immune activation. It will be of interest to analyze whether some of these profiles drive preferentially some morbidities known to be fueled by immune activation, as insulin resistance, atherothrombosis or liver steatosis.

## Introduction

Persistent immune activation (IA) fuels major chronic morbidities, including insulin resistance, metabolic syndrome, diabetes, atherothrombosis, neurocognitive disorders or liver steatosis. A model for IA is HIV-1 infection under efficient combined antiretroviral therapy. In people living with HIV-1, the immune system remains potentially activated, even if viral replication is controled by the treatment^1^. To better characterize IA in this model, we previously measured a series of cell-surface and soluble markers, in 120 efficiently treated HIV patients. A hierarchical clustering analysis identified 5 different IA profiles in these people^2^. To test whether the IA profiles were robust rather than specific to the 120 patients we had analyzed, we recruited 20 more HIV patients with divergent bioclinical characteristics, and performed the hierarchical clustering analysis again^3^. Once more, we observed 5 different IA profiles in these 140 HIV patients. We also analyzed the possibility that these IA profiles were the consequence of different causes. In favour of this model, we found a link between microbial translocation and one of the IA profiles^3^.

The general population, particularly in old age, shares many of the causes of IA with people living with HIV-1. First, we all harbour infectious agents that trigger our immune system^4^. Second, there is a low level of microbial translocation in each individual that increases over time^5^. Third, metabolic disorders that may stimulate the immune system also increase with age^4^. Fourth, as with any senescent cell, immune cells in aging individuals release factors responsible for IA^4^. And last, the efficiency of Treg cells, a CD4+ T cell subpopulation whose function is to downregulate IA, decreases over time^6^. Therefore, we reasoned that a more general population might also present with different IA profiles driven by these various etiologic factors. To test this hypothesis, we looked in the present study for the presence of distinct IA profiles in a general population, and for potential etiologic factors linked to these profiles.

## Materials and methods

### Study design

We recruited 150 adults over 55 years and below 70 years of age, affiliated to the French Social Security system who volunteered for a free health checkup at a Social Security Center in Nîmes, France. Pregnant women, people under immunomodulatory treatment or with diseases likely to modify their immune system were not included. This study was approved by the French Ethics Committee Sud Est IV. All methods were carried out in accordance with the French guidelines and regulations. All individuals had provided written informed consent. The trial was registered on ClinicalTrials.gov under the reference NCT04028882.

### Flow cytometry

Monoclonal antibodies conjugated with fluorescein isothiocyanate (FITC), phycoerythrin (PE), energy-coupled dye (ECD), PE-Cyanine5.5 (PC5.5), PE-Cyanine7 (PC7), allophycocyanine (APC), APCAlexa700, or APCAlexa750 were purchased from Beckman Coulter (Villepinte, France). The antibodies were used in the following combinations; CD57-FITC/CD279-PE/CD45RA-ECD/CD28-PC5.5/CD27-PC7/CD8-APC/CD4-APCAlexa700/CD3-APCAlexa750, CD20-FITC/CD38-PE/HLADR-PC7/CD8-APC/CD4-APCAlexa700/CD3-APCAlexa750, CD57-FITC/CD14-PE/CD56-PC5.5/HLADR-PC7/CD16-APC/CD3-APCAlexa750, CD4-FITC/CD45RA-ECD/CD25-PC7/FoxP3-APC/CD127-APC750. Whole blood collected in EDTA tubes was stained within one hour for 10 mn at room temperature in the dark with the cocktail of antibodies and fixed using Immunoprep Reagent kit (Beckman Coulter) according to the manufacturer’s protocol.

For Treg quantification, cells were first fixed with Reagent 1 of the IntraPrep Permeabilization kit (Beckman Coulter) in the dark, and then stained with the CD4-FITC/CD45RA-ECD/CD25-PC7/CD127-APC750 cocktail of antibodies. Secondly, cells were permeabilized and an anti-FoxP3-APC antibody was added. Finally, red blood cells were lysed using Reagent 2. After one hour, cells were washed with Reagent 3.

Cells were run on a Navios flow cytometer and results were analyzed by using Kaluza^®^ software (Beckman Coulter). A minimum of 20,000 lymphocytes were gated to analyze the subpopulations. We controled the inter-run variability with the same batch of Rainbow 8-peak beads (Beckman Coulter). During the study, no voltage adjustment was necessary to keep the beads into their respective defined targets.

### Soluble immunologic markers in peripheral blood

ELISA was used to quantify soluble TNF receptor I (sTNFRI), soluble CD14 and soluble CD163 (sCD163) (Quantikine, R&D systems, Rennes, France), as well as tissue Plasminogen Activator (tPA) and soluble Endothelial Protein C Receptor (sEPCR) (Asserachrom, Stago, Asnières-sur-Seine, France) in plasma collected in EDTA Vacutainer tubes (Becton Dickinson, Le Pont-de-Claix, France) and frozen. C-Reactive Protein (CRP) and immunoglobulins (Ig) were measured by turbidimetry in plasma collected by the same way. 16s ribosomal bacterial DNA was measured in plasma by quantitative PCR as previously described^7^.

### Statistical analysis

All data were standardized before statistical analysis. Next, a visual assessment of the possibility to cluster the data was made using principal component analysis, and also by seeking a cluster structure in the distance matrix. Next, the Hopkins statistic was calculated, with a value of 1 indicating the highest possibility to cluster the data^8^. Second, we determined the optimal number of clusters using several indexes (e.g., Silhouette^9^, Gap statistic^10^). The majority rule was used to determine the optimal number of clusters. Third, we performed two hierarchical clustering analyses. One clustering analysis was carried for volunteers, using the Euclidian distance to measure the distance between individuals and the other one for markers, using 1-abs (correlation) as a distance. For both of them, Ward’s minimum variance method was used as a linkage method. We then generated a heatmap using the classification of volunteers and markers. We evaluated the appropriateness of the classification through an internal validation test. We used two indexes, based on compactness and separation, (i) the silhouette width which varies between − 1 and 1 representing a wrong and perfect classification^9^, respectively, and (ii) the Dunn index which varies from 0 to infinity and should be maximized^11,12^. In addition, to analyze the significance of the hierarchical classification, we performed a permutation test on volunteer groups. To this aim, we computed the ratio between between-group and within-group variability in the true groups using the *lda* function of the MASS package (MASS_7.3-51.6) of the R software (R version 4.0.2 (2020-06-22)). This function computes the singular values, which give the ratio of the between- and within-group standard deviations on the linear discriminant variables. Their squares are the canonical F-statistics. The sum of all singular values provides the global ratio among between-group and within-group variability. In parallel we generated random groups by permutation of group labels. We repeated the permutation 1000 times and visualized their distribution compared to the ratio obtained from true groups.

In order to characterize each immune profile, a V-test was calculated^13^. The bigger the absolute value of the V-test is, the more characteristic the variable is. All analyses were performed using version 3.6.1 R software (R Development Core Team, A Language and Environment for Statistical Computing, Vienna, Austria, 2016. https://www.R-project.org/).

We used the Mann–Whitney test to compare markers and IA profiles. The links between biomarkers were determined by Spearman rank correlations.

## Results

### Different immune activation profiles may be distinguished in adults

**Figure 1:**
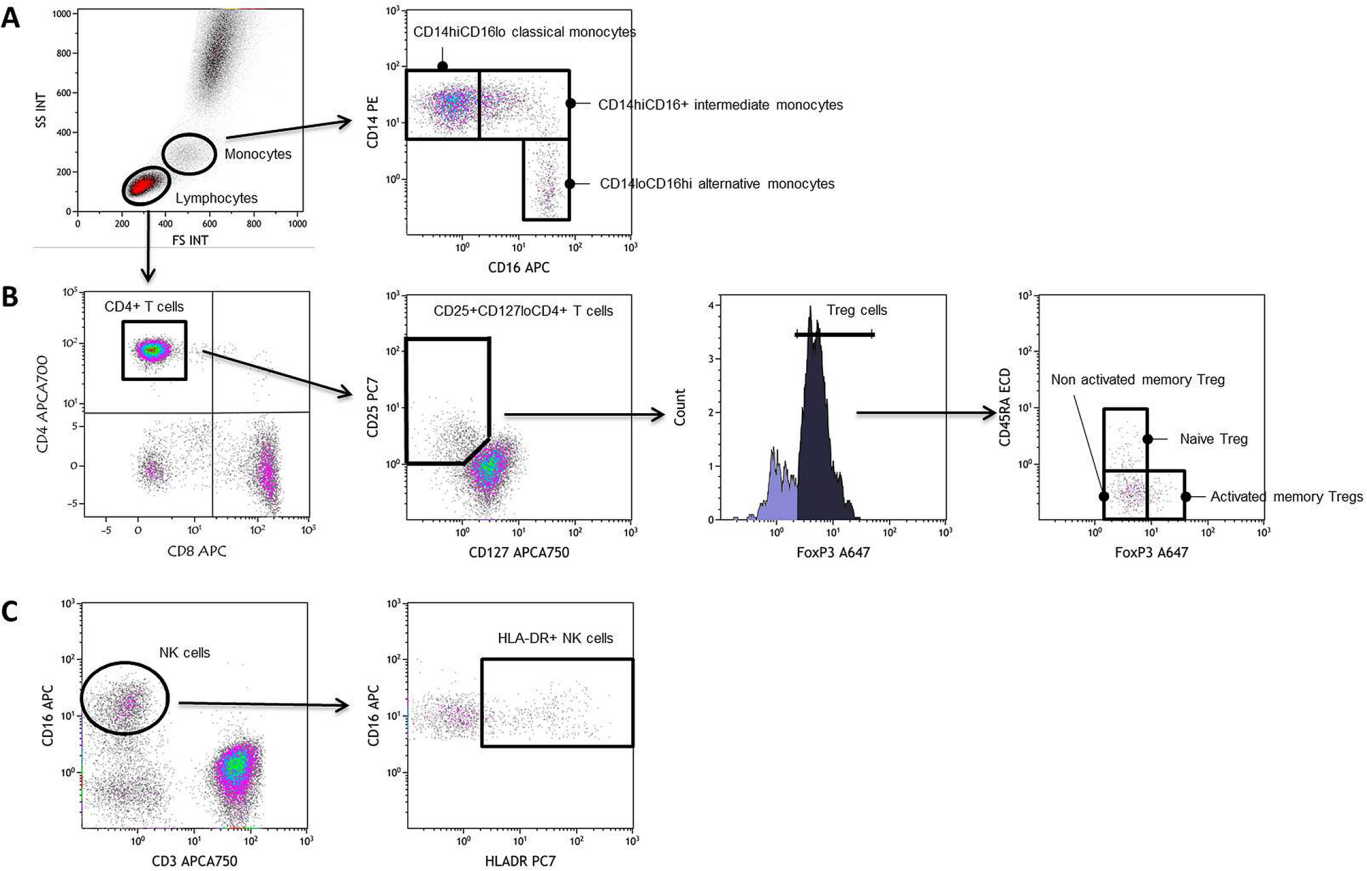
Examples of flow cytometry staining of monocytes **(A)**, CD4+ T cells including Treg cells **(B)**, CD8+ T cells **(B)**, and NK cells **(C)**.

We recruited 74 (49%) women and 76 (51%) men with a mean ± SD age of 62 ± 4 years. We determined the proportions of CD4+ and CD8+ T cells, of naïve (CD27+ CD45RA+), central memory (CD27+ CD45RA-), effector memory (CD27-CD45RA-), and terminally differentiated T cells (CD27-CD45RA+), activated (HLA-DR+ and/or CD38+), exhausted (PD-1+), and senescent (CD57+, eventually CD27- and CD28-) T cells. The percentages of activated (HLA-DR+), dysfunctional (CD56-), and senescent (CD57+) NK cells were also measured. For monocytes, we quantified the classical (CD14hiCD16lo), intermediate (CD14hiCD16+), and alternative CD14loCD16hi) subpopulations. Examples of our gating strategy are shown in Fig. 1. IgG, IgA, IgM, and sCD163 peripheral blood levels were used as markers of B-cell and monocyte activation, respectively. Inflammation was evaluated via sTNFRI and CRP concentrations, and endothelium activation via sEPCR and tPA concentrations in peripheral blood.

**Figure 2:**
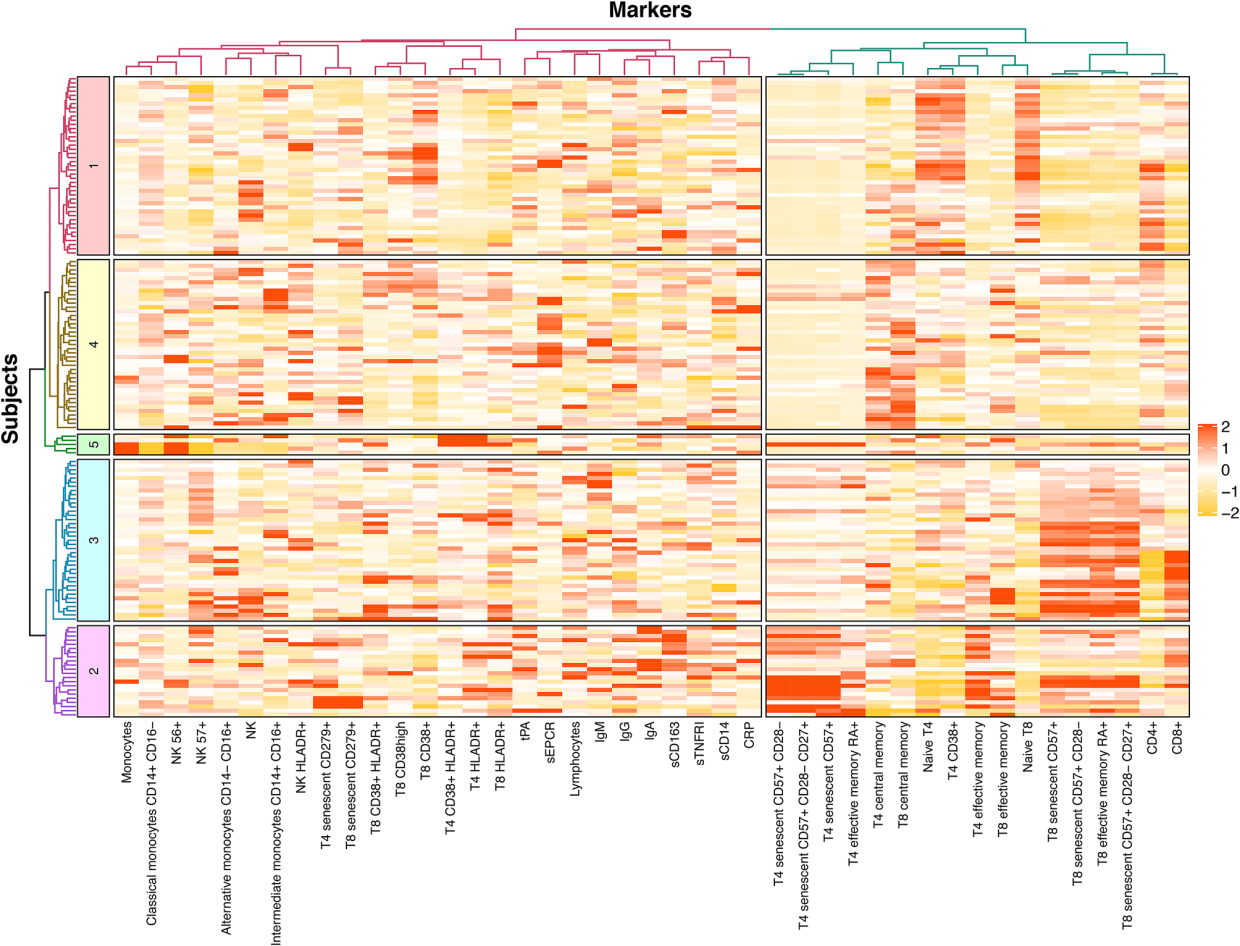
Individuals have different immune activation profiles. Heatmap showing the hierarchical clustering of activation markers (vertical) and volunteers according to their immune activation profile (horizontal). Each immune activation profile is indicated.

**Figure 3:**
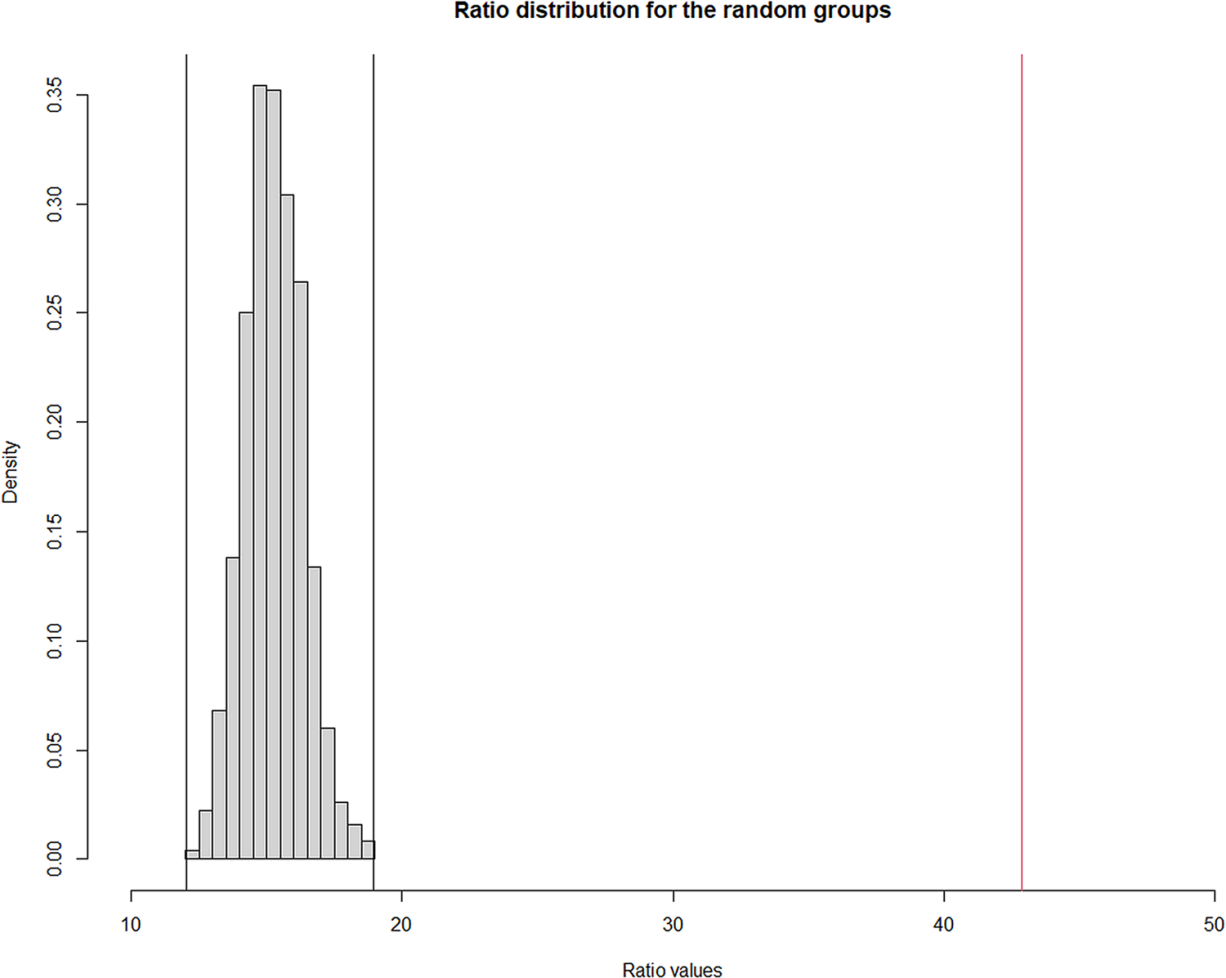
Analyse of the quality of the hierarchical clustering of the volunteers by permutation test. Distribution of the ratio between-group standard deviations: within-group standard deviations for the random groups (grey bars) and the true groups (red line).

**Figure 4:**
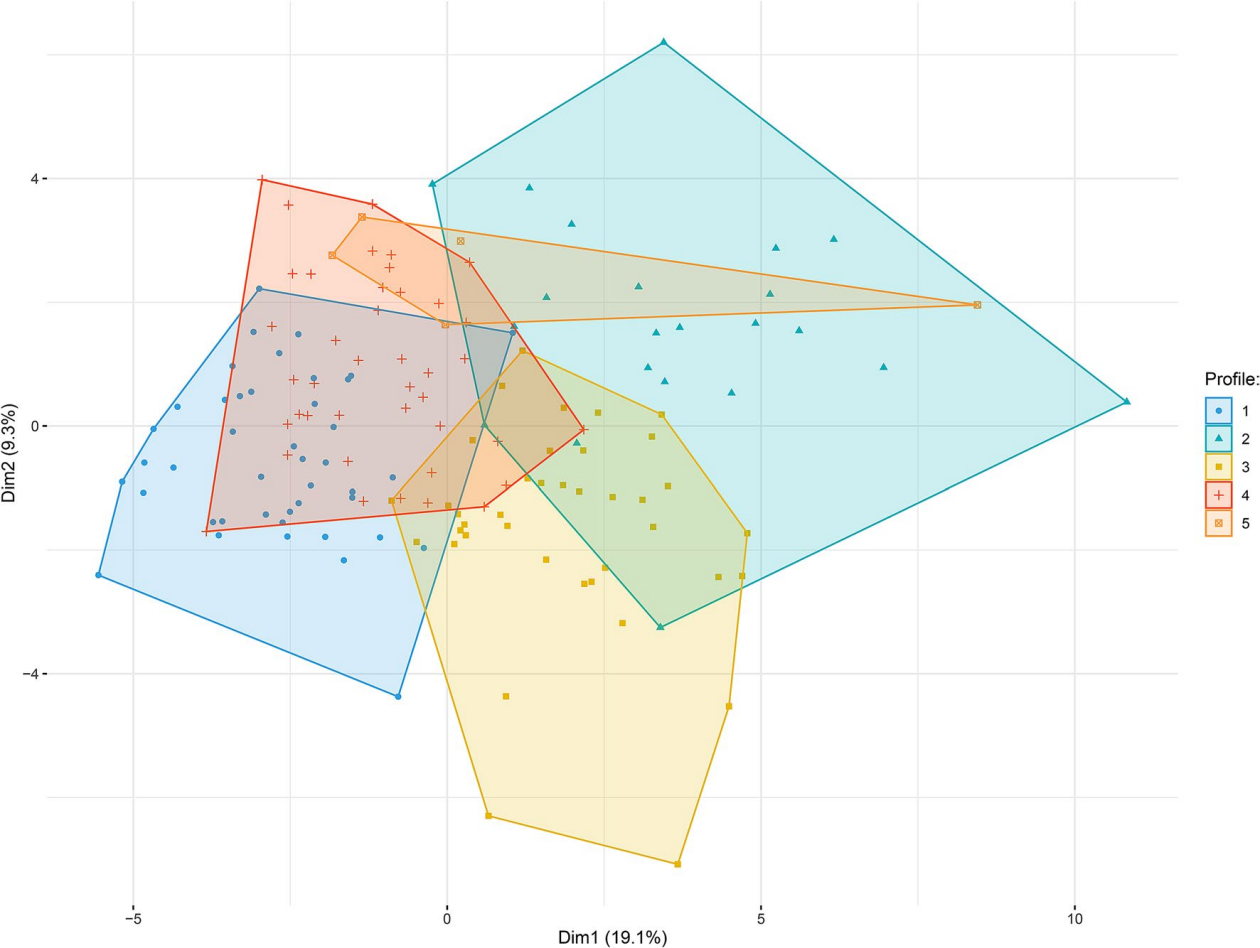
Subjects' map resulting from principal component analysis.

For both markers and individuals, a clustering tendency was observed (Hopkins statistics: 0.68 and 0.73, respectively). We performed two independent hierarchical clustering analyses, one for the activation markers and another one for the volunteers. The number of clusters chosen for markers and donors were 2 and 5, respectively, as they corresponded to the results obtained with the majority of the indexes we tested. Thus, the analysis of volunteers identified 5 groups of individuals presenting with different IA profiles (Fig. 2). Concerning the internal validation step, the Dunn index and the silhouette width were 0.26 and 0.06 for individuals, and 0.39 and 0.12 for markers, respectively. To show that the groups identified by hierarchical classification reflect a true structure of the data, we generated random groups of the same size as the true ones. The within-group and between-group variability allow to investigate the quality of clusters, as “good” clusters are compact (individuals in the same group have similar properties, reflected in a low within-group variability) and far from each other (individuals in different groups present distinct profiles, reflected in high between-group variability). Hence, random groups, or data with no defined clusters, would show a lower ratio between between-group and within-group variability. The distribution of the ratio of the between- and within-group standard deviations on the linear discriminant variables is represented in the Fig. 3. The histogram shows that the between/within ratio is much better for the real groups as the “best” random group exhibits a much lower ratio. Indeed, the median values for the random groups is 15.22 (minimum, 12.04 and maximum, 18.92), whereas it is 42.85 for the true groups, identified by the hierarchical classification (p < 0.001). Next, we computed a Principal Component Analysis of the volunteers (Fig. 4).

### Characterization of the immune activation profiles observed

**Figure 5:**
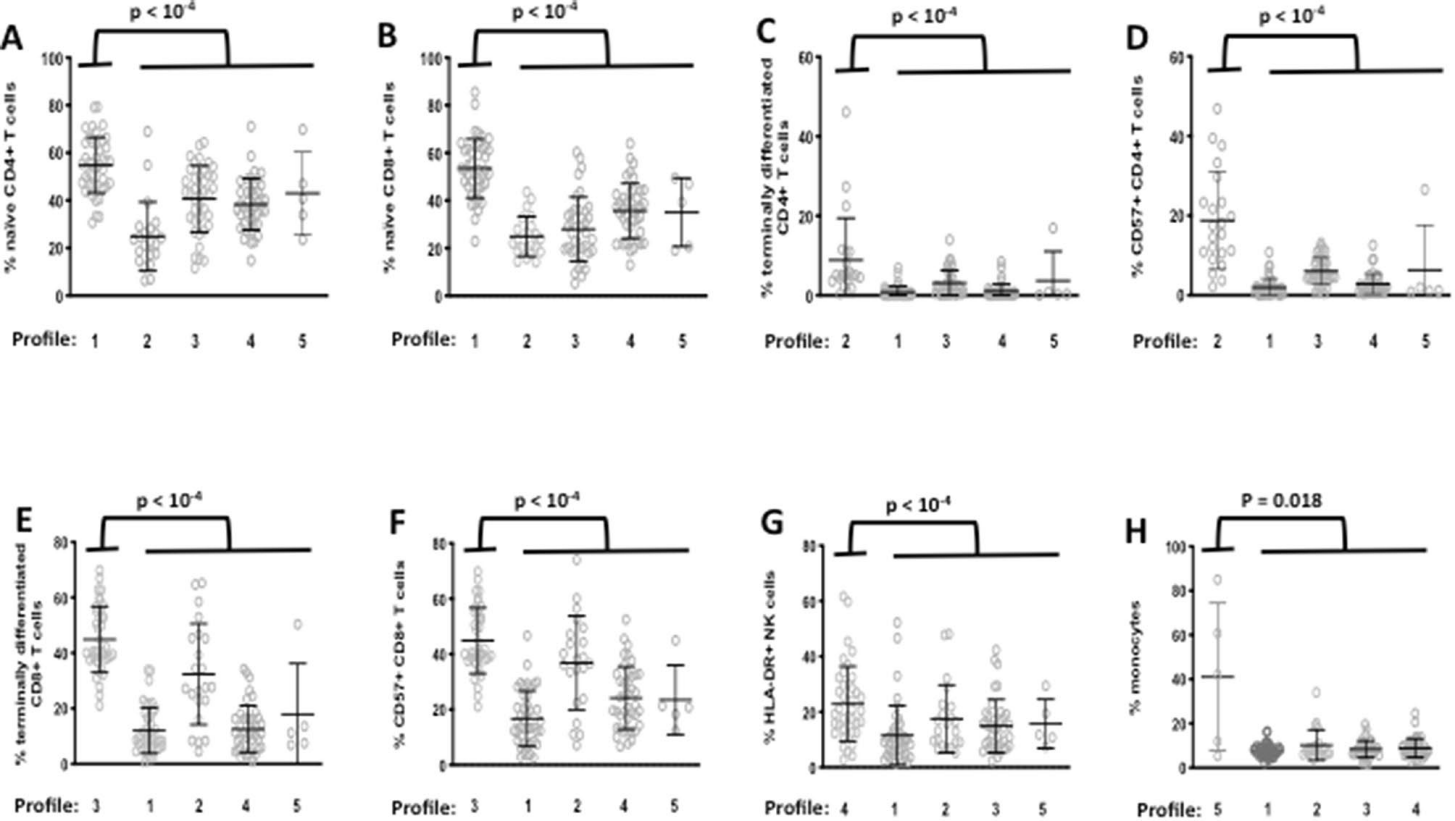
IA profiles are characterized by specific markers. Differences in the level of various activation markers between volunteers with Profile 1 **(A,B)**, 2 **(C,D)**, 3 **(E,F)**, 4 **(G)**, and 5 **(H)**, and the other profiles.

The age, sex, and ethnicity of the individuals are given according to their IA profile in Table 1. There are gender differences in the immune activation profiles (p < 0.001). For each profile, we compared each marker mean with the overall mean, using the V-test (Table 2). Each profile may be characterized by specific markers, comparatively to other profiles. Thus, individuals with Profile 1 had a high mean (± SD) percentage of naïve CD4+ T cells (55 ± 12 versus 37 ± 14, p < 10^–4^, Fig. 5A) and naïve CD8+ T cells (53 ± 12 versus 31 ± 12, p < 10^–4^, Fig. 5B) contrasting with a low frequency of terminally differentiated (1 ± 1 versus 3 ± 6, p < 10^–4^, Fig. 5C) and CD57+ senescent (2 ± 2 versus 7 ± 9, p < 10^–4^, Fig. 5D) CD4+ T cells, as well as of terminally differentiated (12 ± 8 versus 27 ± 18, p < 10^–4^, Fig. 5E) and senescent (17 ± 10 versus 34 ± 16, p < 10^–4^, Fig. 5F) CD8+ T cells. Profiles 2 and 3 were at the opposite end of the spectrum. Profile 2 was characterized by an elevated percentage of terminally differentiated (9 ± 11 versus 2 ± 3, p < 10^–4^, Fig. 5C) and CD57+ senescent (19 ± 12 versus 4 ± 4, p < 10^–4^, Fig. 5D) CD4+ T cells. In Profile 3, it is the CD8+ T cells that were more terminally differentiated (41 ± 12 versus 16 ± 14, p < 10^–4^, Fig. 5E), and senescent (45 ± 12 versus 24 ± 14, p < 10^–4^, Fig. 5F). Profile 4 presented a high proportion of activated NK cells (23 ± 13 versus 14 ± 11, p < 10^–4^, Fig. 5G). Finally, a high proportion of monocytes (41 ± 33 versus 9 ± 4, p = 0.018, Fig. 5H) was noteworthy in Profile 5.

**Table 1:** Volunteers characteristics.

Characteristic	Variable	All profiles	Profile 1 (N = 43)	Profile 2 (N = 22)	Profile 3 (N = 39)	Profile 4 (N = 41)	Profile 5 (N = 5)
Age	Mean (SD)	61.7 (4.3)	61.4 (4.6)	61.8 (4.8)	62.8 (4.3)	61.2 (3.8)	61.0 (2.1)
**Sex** Female Male	% %	48 52	67 33	23 77	59 41	39 61	20 80
**Ethnicity** African Caucasian	% %	8 92	10 90	14 86	10 90	2 98	0 100

**Table 2:** Description of the immune activation profiles using the V-test.

Profile	Variable	Mean (SD) in profile	Overall mean (SD)	V-test
**Profile 1**	Percentage of T8 naive	53.52 (12.38)	37.19 (16.2)	7.8
	Percentage of T4 naive	54.82 (11.7)	41.86 (15.87)	6.32
	Percentage of T8 senescent CD57+	16.72 (10.01)	29.26 (16.42)	− 5.91
	Percentage of T4 CD38	62.22 (13.3)	50.82 (15.71)	5.61
	Percentage of T8 HLA-DR+	27.12 (13.01)	39.5 (17.48)	− 5.48
	Percentage of T8 senescent CD57+CD28−	13.71 (8.91)	24.65 (16.03)	− 5.28
	Percentage of T8 senescent CD57+CD28−CD27−	9.76 (6.98)	19.93 (15.68)	− 5.02
	Percentage of T8 terminally differentiated	12.06 (8.21)	22.98 (17.32)	− 4.88
	Percentage of T4 effector memory	6.06 (2.97)	11.47 (9.16)	− 4.58
	Percentage of T4 HLA-DR+	9.61 (4.63)	15.9 (11.06)	− 4.4
	Percentage of T8 CD38+	37.54 (13.11)	29.94 (13.49)	4.36
	Percentage of T8 effector memory	5.42 (2.87)	9.34 (7.17)	− 4.23
	Percentage of T4	72.88 (9.32)	66.77 (11.54)	4.1
	Percentage of T4 senescent CD57+	1.96 (2.25)	5.88 (7.87)	− 3.86
	Percentage of T8	23.4 (8.33)	28.45 (10.16)	− 3.85
	Percentage of T4 senescent CD57+CD28−	1.14 (1.77)	3.83 (5.81)	− 3.58
	Percentage of T4 senescent CD57+CD28−CD27−	0.99 (1.69)	3.61 (5.8)	− 3.5
	Percentage of T4 central memory	38.3 (9.66)	43.62 (11.86)	− 3.47
	Percentage of NK CD57+	42.16 (14.66)	49.21 (16.36)	− 3.33
	Percentage of NK HLA-DR+	11.58 (10.63)	16.55 (12.11)	− 3.18
	Percentage of NK CD56−	9.55 (6.17)	16.4 (17.15)	− 3.09
	Percentage of T8 CD38+HLA-DR+	8.37 (4.69)	11.63 (8.79)	− 2.87
	Percentage of T4 CD38+HLA-DR+	3.92 (1.54)	5.36 (3.98)	− 2.81
	Percentage of T4 terminally differentiated	0.82 (1.43)	2.75 (5.37)	− 2.77
	Percentage of classical monocytes CD14hiCD16lo	83.32 (6.84)	79.9 (14.37)	1.84
	Percentage of monocytes	7.6 (2.84)	9.67 (9.06)	− 1.77
	IgM	1.09 (0.53)	1.26 (0.73)	− 1.75
	sEPCR	126.89 (69.32)	146.45 (89.39)	− 1.69
	Percentage of T4 exhausted CD279+	8.72 (4.94)	10.57 (8.83)	− 1.62
	sCD163	625.84 (287.63)	683.64 (283.54)	− 1.58
	Percentage of alternative monocytes CD14loCD16hi	4.64 (3.1)	5.53 (4.84)	− 1.43
	CRP	2.16 (2.49)	2.83 (3.68)	− 1.41
	sTNFRI	1.48 (0.26)	1.55 (0.39)	− 1.39
	sCD14	1708.43 (204.85)	1657.59 (298.19)	1.32
	IgA	2.32 (1)	2.5 (1.15)	− 1.27
	Percentage of intermediate monocytes CD14hiCD16+	9.09 (4.63)	9.99 (6.73)	− 1.04
	tPA	9.75 (4.93)	10.51 (6.09)	− 0.96
	Percentage of T8 central memory	28.99 (10.49)	30.51 (12.69)	− 0.92
	Lymphocytes	1838.49 (556.16)	1904.7 (593.2)	− 0.86
	Percentage of T8 CD38hi	2.8 (2.05)	2.66 (2.79)	0.41
	Percentage of T8 exhausted CD279+	16.24 (10.09)	15.93 (12.13)	0.2
	IgG	9.91 (2.07)	9.93 (2.07)	− 0.07
	Percentage of NK	8.21 (5.89)	8.22 (5.34)	− 0.02
**Profile 2**	Percentage of T4 senescent CD57+	18.76 (12.2)	5.88 (7.87)	8.28
	Percentage of T4 senescent CD57+CD28−	13.03 (8.45)	3.83 (5.81)	8.01
	Percentage of T4 senescent CD57+CD28−CD27−	12.6 (8.73)	3.61 (5.8)	7.85
	Percentage of T4 effector memory	23.87 (13.55)	11.47 (9.16)	6.85
	Percentage of T4 terminally differentiated	8.87 (10.58)	2.75 (5.37)	5.78
	Percentage of T4 naive	24.9 (14.44)	41.86 (15.87)	− 5.41
	Percentage of T4 CD38+	34.39 (12.73)	50.82 (15.71)	− 5.29
	Percentage of T4 exhausted CD279+	19.29 (17.63)	10.57 (8.83)	5
	sCD163	940.28 (397.75)	683.64 (283.54)	4.58
	IgA	3.39 (1.95)	2.5 (1.15)	3.9
	Percentage of T8 naive	24.93 (8.32)	37.19 (16.2)	− 3.83
	Percentage of T8 effector memory	14.74 (9.67)	9.34 (7.17)	3.81
	Percentage of T8 CD38+	21.43 (9.64)	29.94 (13.49)	− 3.19
	Percentage of T4 HLA-DR+	22.51 (11.26)	15.9 (11.06)	3.03
	Percentage of T8 terminally differentiated	32.35 (18.21)	22.98 (17.32)	2.74
	Percentage of T4	60.9 (10.24)	66.77 (11.54)	− 2.57
	tPA	13.58 (6.24)	10.51 (6.09)	2.55
	Percentage of T8 HLA-DR+	47.89 (18.53)	39.5 (17.48)	2.43
	Percentage of T8	33.22 (10.96)	28.45 (10.16)	2.37
	Percentage of T8 senescent CD57+	36.86 (16.98)	29.26 (16.42)	2.34
	Lymphocytes	2179.32 (763.19)	1904.7 (593.2)	2.34
	IgG	10.74 (2.16)	9.93 (2.07)	1.98
	Percentage of T8 exhausted CD279+	20.62 (18.36)	15.93 (12.13)	1.96
	Percentage of T8 senescent CD57+CD28−CD27−	25.94 (17.09)	19.93 (15.68)	1.94
	Percentage of alternative monocytes CD14loCD16hi	3.93 (2.58)	5.53 (4.84)	− 1.67
	Percentage of NK CD57+	54.4 (15.15)	49.21 (16.36)	1.6
	Percentage of T8 senescent CD57+CD28−	29.67 (17.09)	24.65 (16.03)	1.58
	sTNFRI	1.67 (0.42)	1.55 (0.39)	1.57
	Percentage of classical monocytes CD14hiCD16lo	84.32 (7.23)	79.9 (14.37)	1.55
	Percentage of T4 central memory	40.32 (15.23)	43.62 (11.86)	− 1.41
	sCD14	1725.62 (274.65)	1657.59 (298.19)	1.15
	Percentage of T8 CD38hi	2.06 (1.83)	2.66 (2.79)	− 1.09
	Percentage of T8 central memory	28.14 (12.79)	30.51 (12.69)	− 0.95
	Percentage of intermediate monocytes CD14hiCD16+	9.08 (6.7)	9.99 (6.73)	− 0.69
	Percentage of T4 CD38+HLA-DR+	4.99 (2.1)	5.36 (3.98)	− 0.47
	Percentage of NK CD56–	14.85 (12)	16.4 (17.15)	− 0.46
	IgM	1.32 (0.77)	1.26 (0.73)	0.43
	Percentage of T8 CD38+HLA-DR+	10.94 (6.19)	11.63 (8.79)	− 0.4
	Percentage of NK HLA-DR+	17.48 (12.22)	16.55 (12.11)	0.39
	Percentage of monocytes	10.23 (6.9)	9.67 (9.06)	0.31
	CRP	3.01 (3.65)	2.83 (3.68)	0.26
	Percentage of NK	8 (4.58)	8.22 (5.34)	− 0.21
	sEPCR	148.49 (81.15)	146.45 (89.39)	0.12
**Profile 3**	Percentage of T8 terminally differentiated	41.34 (12.49)	22.98 (17.32)	7.67
	Percentage of T8 senescent CD57+CD28−CD27−	36.46 (12.84)	19.93 (15.68)	7.63
	Percentage of T8 senescent CD57+CD28−	41.04 (11.73)	24.65 (16.03)	7.4
	Percentage of T8 senescent CD57+	44.89 (11.88)	29.26 (16.42)	6.89
	Percentage of T8 central memory	20.8 (6.07)	30.51 (12.69)	− 5.53
	Percentage of T4	60.04 (13.06)	66.77 (11.54)	− 4.22
	Percentage of T8	34.36 (10.92)	28.45 (10.16)	4.21
	Percentage of T8 CD38+HLA-DR+	16.75 (12.82)	11.63 (8.79)	4.21
	Percentage of T8 naive	28 (13.42)	37.19 (16.2)	− 4.11
	Percentage of NK CD57+	56.9 (14.01)	49.21 (16.36)	3.4
	Percentage of T8 HLA-DR+	46.84 (18.3)	39.5 (17.48)	3.04
	Percentage of alternative monocytes CD14loCD16hi	7.41 (6.98)	5.53 (4.84)	2.81
	IgM	1.51 (0.75)	1.26 (0.73)	2.5
	Percentage of T8 exhausted CD279+	12.34 (9.18)	15.93 (12.13)	− 2.14
	sEPCR	122.18 (42.08)	146.45 (89.39)	− 1.96
	sCD14	1594.01 (373.68)	1657.59 (298.19)	− 1.54
	sCD163	624.32 (191.54)	683.64 (283.54)	− 1.51
	Percentage of NK CD56−	12.89 (7.33)	16.4 (17.15)	− 1.48
	Percentage of T4 effector memory	13.13 (8.14)	11.47 (9.16)	1.31
	CRP	2.17 (2.75)	2.83 (3.68)	− 1.28
	Lymphocytes	2007.18 (531.31)	1904.7 (593.2)	1.25
	tPA	9.63 (4.39)	10.51 (6.09)	− 1.04
	Percentage of monocytes	8.42 (3.78)	9.67 (9.06)	− 1
	Percentage of NK HLA-DR+	14.92 (9.6)	16.55 (12.11)	− 0.97
	IgA	2.36 (0.75)	2.5 (1.15)	− 0.93
	Percentage of T4 exhausted CD279+	9.69 (5.54)	10.57 (8.83)	− 0.72
	Percentage of T4 CD38+HLA-DR+	5.75 (2.47)	5.36 (3.98)	0.7
	Percentage of T8 CD38hi	2.92 (4.36)	2.66 (2.79)	0.67
	IgG	10.09 (1.97)	9.93 (2.07)	0.56
	Percentage of T4 naive	40.68 (13.93)	41.86 (15.87)	− 0.54
	Percentage of T8 effector memory	9.86 (7.72)	9.34 (7.17)	0.53
	Percentage of T4 terminally differentiated	3.11 (3.19)	2.75 (5.37)	0.49
	sTNFRI	1.57 (0.33)	1.55 (0.39)	0.47
	Percentage of T8 CD38+	30.71 (12.99)	29.94 (13.49)	0.41
	Percentage of T4 central memory	43.08 (10.12)	43.62 (11.86)	− 0.33
	Percentage of T4 senescent CD57+CD28−	4.07 (2.91)	3.83 (5.81)	0.3
	Percentage of T4 senescent CD57+CD28−CD27−	3.81 (2.9)	3.61 (5.8)	0.25
	Percentage of classical monocytes CD14hiCD16lo	79.45 (11.29)	79.9 (14.37)	− 0.23
	Percentage of T4 CD38+	51.29 (13.08)	50.82 (15.71)	0.22
	Percentage of T4 senescent CD57+	6.1 (3.32)	5.88 (7.87)	0.2
	Percentage of intermediate monocytes CD14hiCD16+	10.15 (6.31)	9.99 (6.73)	0.17
	Percentage of T4 HLA-DR+	15.76 (6.71)	15.9 (11.06)	− 0.09
	Percentage of NK	8.17 (5.42)	8.22 (5.34)	− 0.07
**Profile 4**	Percentage of T8 central memory	42.14 (10.29)	30.51 (12.69)	6.86
	Percentage of T4 central memory	51.7 (9.52)	43.62 (11.86)	5.1
	Percentage of T8 terminally differentiated	12.58 (8.44)	22.98 (17.32)	− 4.5
	Percentage of NK HLA-DR+	22.92 (13.49)	16.55 (12.11)	3.94
	Percentage of T8 senescent CD57+CD28−CD27−	12.05 (8.37)	19.93 (15.68)	− 3.76
	sEPCR	187.69 (127.81)	146.45 (89.39)	3.45
	Percentage of T4 senescent CD57+CD28−	1.32 (2.19)	3.83 (5.81)	− 3.24
	Percentage of T4 senescent CD57+CD28−CD27−	1.16 (2.17)	3.61 (5.8)	− 3.16
	Percentage of T8 senescent CD57+CD28−	18.37 (11.17)	24.65 (16.03)	− 2.93
	Percentage of T4 senescent CD57+	2.83 (2.54)	5.88 (7.87)	− 2.9
	Lymphocytes	1704.27 (539)	1904.7 (593.2)	− 2.53
	Percentage of T4 terminally differentiated	1.01 (1.82)	2.75 (5.37)	− 2.42
	Percentage of T8 senescent CD57+	24.16 (11.32)	29.26 (16.42)	− 2.32
	Percentage of NK CD56−	21.7 (20.08)	16.4 (17.15)	2.32
	CRP	3.92 (5.13)	2.83 (3.68)	2.23
	Percentage of T4 effector memory	8.93 (3.47)	11.47 (9.16)	− 2.08
	Percentage of T8	25.83 (6.88)	28.45 (10.16)	− 1.93
	Percentage of T4	69.68 (8.17)	66.77 (11.54)	1.89
	Percentage of intermediate monocytes CD14hiCD16+	11.61 (8.2)	9.99 (6.73)	1.8
	Percentage of T8 CD38+	26.9 (12.4)	29.94 (13.49)	− 1.69
	Percentage of T4 naive	38.36 (10.86)	41.86 (15.87)	− 1.65
	IgG	9.54 (2.08)	9.93 (2.07)	− 1.41
	Percentage of T4 exhausted CD279+	8.92 (4.47)	10.57 (8.83)	− 1.4
	IgA	2.31 (0.78)	2.5 (1.15)	− 1.27
	Percentage of T4 CD38+	48.55 (12.48)	50.82 (15.71)	− 1.08
	Percentage of T8 CD38+HLA-DR+	10.41 (6.3)	11.63 (8.79)	− 1.04
	Percentage of NK	8.9 (5.15)	8.22 (5.34)	0.95
	Percentage of T8 naive	35.64 (11.54)	37.19 (16.2)	− 0.72
	IgM	1.19 (0.86)	1.26 (0.73)	− 0.67
	Percentage of monocytes	8.9 (4.19)	9.67 (9.06)	− 0.64
	Percentage of NK CD57+	50.54 (12.77)	49.21 (16.36)	0.61
	sCD14	1634.07 (315.53)	1657.59 (298.19)	− 0.59
	Percentage of T4 CD38+HLA-DR+	5.07 (1.91)	5.36 (3.98)	− 0.56
	Percentage of T8 HLA-DR+	40.59 (12.26)	39.5 (17.48)	0.47
	Percentage of alternative monocytes CD14loCD16hi	5.82 (4.17)	5.53 (4.84)	0.46
	Percentage of T4 HLA-DR+	15.31 (5.43)	15.9 (11.06)	− 0.39
	sCD163	671.56 (216.79)	683.64 (283.54)	− 0.32
	Percentage of T8 effector memory	9.64 (6.14)	9.34 (7.17)	0.31
	tPA	10.71 (7.88)	10.51 (6.09)	0.25
	Percentage of classical monocytes CD14hiCD16lo	80.24 (10.75)	79.9 (14.37)	0.18
	Percentage of T8 exhausted CD279+	16.2 (12.07)	15.93 (12.13)	0.17
	Percentage of T8 CD38hi	2.72 (2.06)	2.66 (2.79)	0.16
	sTNFRI	1.54 (0.52)	1.55 (0.39)	− 0.14
**Profile 5**	Percentage of monocytes	41.04 (33.36)	9.67 (9.06)	7.85
	Percentage of T4 CD38+HLA-DR+	18.86 (13.95)	5.36 (3.98)	7.68
	Percentage of classical monocytes CD14hiCD16lo	31.83 (37.9)	79.9 (14.37)	− 7.58
	Percentage of NK CD56−	65.91 (35.69)	16.4 (17.15)	6.54
	Percentage of T4 HLA-DR+	46.66 (33.09)	15.9 (11.06)	6.3
	Percentage of NK CD57+	16.1 (19.1)	49.21 (16.36)	− 4.59
	Percentage of NK	4.23 (4.07)	8.22 (5.34)	− 1.7
	IgG	8.48 (1.54)	9.93 (2.07)	− 1.58
	Percentage of T4 CD38+	40.04 (14.8)	50.82 (15.71)	− 1.56
	Percentage of T8 CD38+	20.92 (11.12)	29.94 (13.49)	− 1.52
	Percentage of alternative monocytes CD14loCD16hi	3.16 (5.94)	5.53 (4.84)	− 1.11
	Percentage of T8 effector memory	12.76 (6.82)	9.34 (7.17)	1.08
	IgA	2.99 (1.28)	2.5 (1.15)	0.95
	Percentage of T8 CD38hi	1.55 (0.92)	2.66 (2.79)	− 0.9
	IgM	0.97 (0.24)	1.26 (0.73)	− 0.9
	Percentage of intermediate monocytes CD14hiCD16+	7.37 (11.31)	9.99 (6.73)	− 0.88
	Percentage of T8 senescent CD57+	23.5 (12.51)	29.26 (16.42)	− 0.79
	Lymphocytes	2110 (431.86)	1904.7 (593.2)	0.78
	Percentage of T8 central memory	34.27 (13.99)	30.51 (12.69)	0.67
	Percentage of T8 terminally differentiated	17.9 (18.36)	22.98 (17.32)	− 0.67
	tPA	8.76 (6.84)	10.51 (6.09)	− 0.65
	CRP	3.86 (3.34)	2.83 (3.68)	0.64
	Percentage of T8 senescent CD57+CD28−	20.29 (13.26)	24.65 (16.03)	− 0.62
	Percentage of T4 senescent CD57+CD28−CD27−	5.14 (10.52)	3.61 (5.8)	0.6
	Percentage of T4 senescent CD57+CD28−	5.26 (10.51)	3.83 (5.81)	0.56
	sCD163	613.39 (191.72)	683.64 (283.54)	− 0.56
	Percentage of T4 exhausted CD279+	8.51 (5.55)	10.57 (8.83)	− 0.53
	Percentage of T8 senescent CD57+CD28−CD27−	16.71 (14.99)	19.93 (15.68)	− 0.47
	Percentage of CD8	26.38 (8.14)	28.45 (10.16)	− 0.46
	Percentage of T8 exhausted CD279+	18.34 (11.25)	15.93 (12.13)	0.45
	Percentage of T8 HLA-DR+	42.78 (23.01)	39.5 (17.48)	0.43
	sTNFRI	1.48 (0.3)	1.55 (0.39)	− 0.41
	Percentage of T4 terminally differentiated	3.68 (7.41)	2.75 (5.37)	0.4
	sCD14	1610 (228.56)	1657.59 (298.19)	− 0.36
	Percentage of T4	68.56 (9.39)	66.77 (11.54)	0.35
	Percentage of T4 central memory	41.9 (11.33)	43.62 (11.86)	− 0.33
	Percentage of T8 naive	35.08 (14.41)	37.19 (16.2)	− 0.3
	Percentage of T8 CD38+HLA-DR+	12.73 (9.87)	11.63 (8.79)	0.28
	sEPCR	156.75 (70.83)	146.45 (89.39)	0.26
	Percentage of T4 naive	43.03 (17.31)	41.86 (15.87)	0.17
	Percentage of NK HLA-DR+	15.72 (8.84)	16.55 (12.11)	− 0.16
	Percentage of T4 senescent CD57+	6.3 (11.33)	5.88 (7.87)	0.12
	Percentage of T4 effector memory	11.38 (8.06)	11.47 (9.16)	− 0.02

Markers are presented from the most to the least characteristic by profile.

### Links between immune activation profiles and etiologic factors

**Figure 6:**
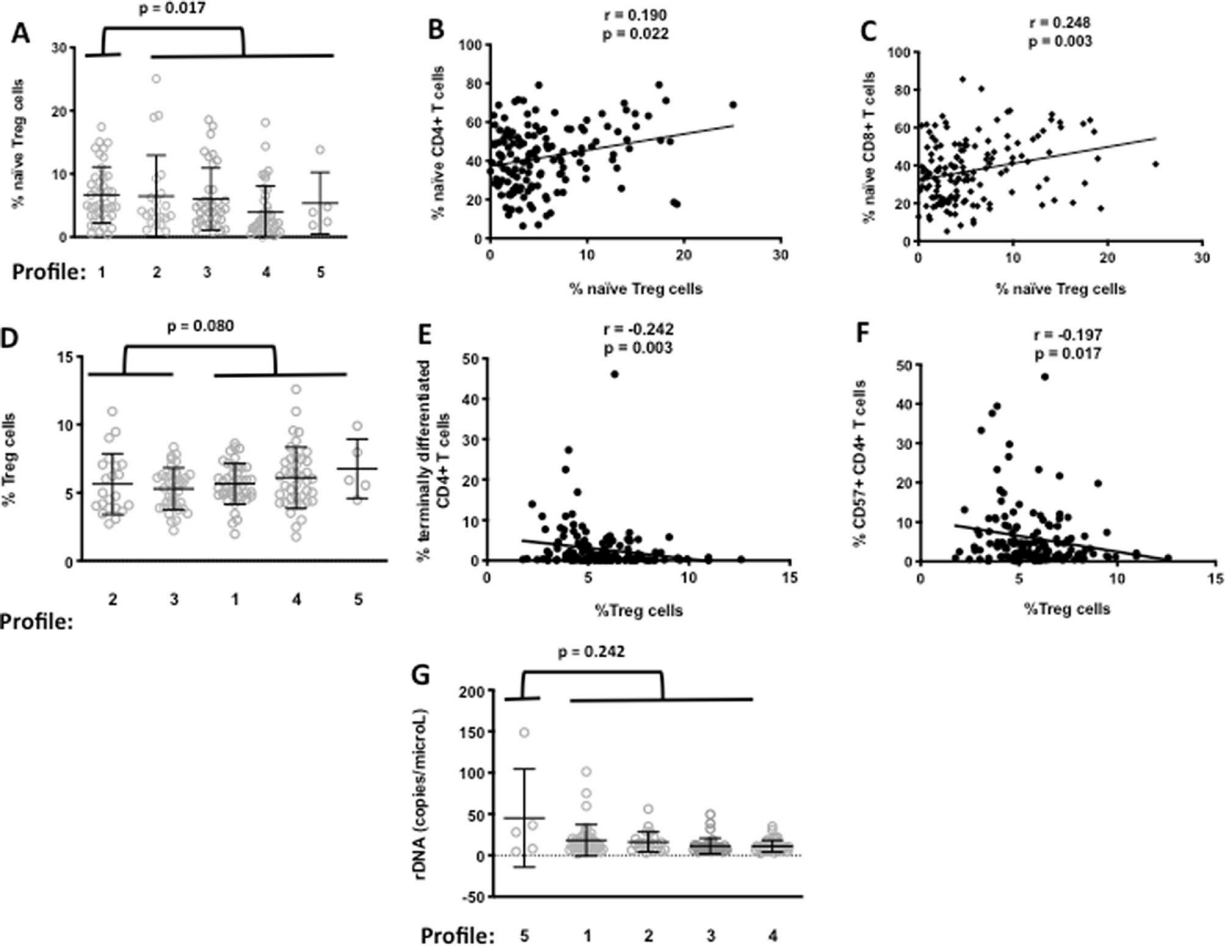
Immune activation Profiles 1, 2, 3, and 5 may be characterized by specific etiologic factors. Naïve Treg frequencies in Profile 1 as compared with the other profiles **(A)**, and correlations with naïve CD4+ T cell **(B)** and naïve CD8+ T cell **(C) **subpopulations. Treg frequencies in Profiles 2 and 3 comparatively to Profiles 1, 4, and 5 **(D)**, and correlations with terminally differentiated **(E)** and senescent **(F)** CD4+ T cells. Circulating bacterial DNA levels in Profile 5 in comparison with the other profiles **(G)**.

In Humans, various factors may be responsible for chronic immune activation. Thus, for instance, a deficiency in the mechanisms responsible for downregulating IA may be responsible for an overactivity of the immune system. Microbial translocation, the entry into our organism of microbial products originating from our microbiota, is another potential cause of IA. In a given individual, only some of these etiologic factors may be at work, e.g., immune dysregulation, but not microbial translocation. Therefore, we tested the hypothesis that the IA profiles that we unraveled might be fueled by specific causes. To this aim, we searched for correlations between potential etiologic factors and each profile. Treg is a CD4+ T cell subpopulation playing a major role in IA downregulation ^14^. Therefore, we measured the proportions and numbers of total Treg (CD4+ CD25+ FOXP3+ CD127lo), naïve Treg (CD4+ CD25+ FOXP3+ CD45RA+CD127lo), and memory Treg, activated (CD4+CD25hiFOXP3hiCD45RA-CD127lo) or not (CD4+CD25+FOXP3+CD45RA-CD127lo) in each individual. As compared with the other profiles, Profile 1, characterized by a low level of differentiated and senescent T cells, had a high percentage of naïve Treg (6.6 ± 4.4 versus 5.3 ± 5.0, p = 0.017, Fig. 6A), a reservoir of cells able to inhibit IA. Moreover, this percentage was linked to the proportions of naïve CD4+ T cells (r = 0.190, p = 0.022, Fig. 6B) and of naïve CD8+ T cells (r = 0.248, p = 0.003, Fig. 6C) in the whole population. By contrast, Profiles 2 and 3, characterized by a high level of differentiated and senescent T cells, tended to have a low percentage of Treg (5.4 ± 1.8 versus 5.9 ± 1.9, p = 0.080, Fig. 6D), in comparison with Profiles 1, 4, and 5. In addition, this percentage was negatively correlated with the proportions of terminally differentiated (r = -0.242, p = 0.003, Fig. 6E) and senescent (r = -0.197, p = 0.017, Fig. 6F) CD4+ T cells in the whole population.

Another potential cause of IA is the intensity of microbial translocation which increases with age^5^. A direct marker of microbial translocation is the presence of bacterial DNA in the circulation quantified by PCR targeting conserved sequences of the 16s ribosomal gene (rDNA). Strikingly, we observed a high, although not significant, level of rDNA in Profile 5 people (45 ± 59 versus 14 ± 13 copies/mL, p = 0.242, Fig. 6G), comparatively to the other volunteers.

## Discussion

In this study, we have shown that various IA profiles may be distinguished using an unsupervised learning method in a population of adults volunteering for a health checkup. We revealed 5 distinct IA profiles that may be characterized according to their levels of CD4+ T cell, CD8+ T cell, NK cell and monocyte frequency, activation, and/or differentiation. One profile, Profile 1, has the lowest level of differentiated and senescent T cells, of activated NK cells, and the lowest percentage of monocytes. It may therefore be considered as the less activated profile. Of note, it is the group with the highest percentage of women. Profiles 2 and 3 are remarkable by their reduced percentages of naïve T cells and their elevated percentages of differentiated and senescent T cells. These profiles may therefore be considered as the T cell activated profiles. In Profile 4 it is the NK cells that are activated, and in Profile 5 it is the frequency of circulating monocytes that is noteworthy.

A second finding of this study is that some of these IA profiles are linked to potential causes of IA. Thus, Profile 5 individuals, characterized by a high frequency of peripheral blood monocytes, have a high circulating bacterial DNA load. Yet, the difference in rDNA level between Profile 5 and the other profiles was not significant, probably due to the small number of participants with this Profile (n = 5). Yet, this link between microbial translocation and the frequency of circulationg monocytes is in line with the observation that bacterial products may boost monopoiesis via TLR signaling^15^. Profiles 2 and 3 which are particular because of their high degree of CD4+ T cell and CD8+ T cell differentiation and senescence, tend to have low percentages of Treg. As Treg are known to interrupt the process of T cell activation^16^, these low Treg levels might participate in the increased T cell differentiation and senescence specific to these profiles. This hypothesis is supported by our observation of an inverse correlation between the percentage of Treg cells on one hand, and of differentiated and senescent CD4+ T cells on the other hand. Conversely, the high percentage of naïve Treg cells in Profile 1 may at least partly explain the low level of T cell differentiation and senescence in that profile.

One of the limitations of our study is that it is cross-sectional, highlighting only correlations. Further analysis is needed to definitively establish causative links between etiologic factors and IA profiles. Also, additional etiologic factors, different from the one we tested, could shape the IA profiles, as for instance the genetic background and the clinical history. Moreover, the stability over time of IA profiles has to be verified. Our study is also limited by the technology we used. Thus, we did not test the functionality of the various immune cell subpopulations we analyzed. The immune phenotyping could also be more precise by using single-cell transcriptomics analysis, high dimensional flow cytometry or CyTOF. On the other hand, our ultimate goal is to identify a simple signature of easily measurable markers characteristic of immune activation profiles that could fuel immune activation-induced morbidities. This goal may be achievable in routine with the tools we used.

Globally, we show that different IA profiles may be distinguished in a general population, and that some of these profiles are linked to potential etiologic factors such as Treg frequency and microbial translocation.

We propose a model where in each individual one or a few specific causes of IA shape a specific IA profile. Of interest, the different IA profiles we describe here may fuel different morbidities among those driven by IA, as insulin resistance, atherothrombosis or liver steatosis for instance, In this hypothesis, immune profiling might help to tailor the prevention and the screening of these IA-induced diseases. Moreover, deciphering soluble immune factors favoring each of these morbidities might open the way to new therapeutic strategies. In the near future, the immune activation profile of each individual might be identified via a simple signature of a reduced number of activation markers easily measurable, and this immune activation profile might predict the chronic morbidiites this individual is at risk of developing.
